# Reliability of the modified child and adolescent physical activity and nutrition survey, physical activity (CAPANS-PA) questionnaire among chinese-australian youth

**DOI:** 10.1186/1471-2288-11-122

**Published:** 2011-08-25

**Authors:** Claudia Strugnell, Andre Renzaho, Kate Ridley, Cate Burns

**Affiliations:** 1 School of Exercise and Nutrition Sciences, Deakin University, 221 Burwood Hwy, Burwood, Victoria 3125, Australia; 2 WHO Collaborating Centre for Obesity Prevention, Deakin University, 221 Burwood Hwy, Burwood, Victoria 3125, Australia; 3 School of Education, Flinders University, GPO Box 2100, Adelaide, South Australia 5001, Australia

## Abstract

**Background:**

Evidence suggests that differences exist in physical activity (PA) participation among Culturally and Linguistically Diverse (CALD) children and adolescents. It is possible that these differences could be influenced by variations in measurement technique and instrument reliability. However, culturally sensitive instruments for examining PA behaviour among CALD populations are lacking. This study tested the reliability of the Child and Adolescent Physical Activity and Nutrition Survey (CAPANS-PA) recall questionnaire among a sample of Chinese-Australian youth.

**Methods:**

The psychometric property of the CAPANS-PA questionnaire was examined among a sample of 77 Chinese-Australian youth (aged 11 - 14 y) who completed the questionnaire twice within 7 days. Test-retest reliability of individual items and scales within the CAPANS-PA questionnaire was determined using Kappa statistics for categorical variables and intraclass correlation coefficients (ICC) for continuous variables.

**Results:**

The CAPANS-PA questionnaire demonstrated acceptable test-retest reliability for frequency and duration of time spent in weekly Moderate to Vigorous Physical Activity (MVPA) (ICC ≥ 0.70) for all participants. Test-retest reliability for time spent in weekly sedentary activities was acceptable for females (ICC = 0.82) and males (ICC = 0.72).

**Conclusions:**

The results suggest the CAPANS-PA questionnaire provides reliable estimates for type, frequency and duration of MVPA participation among Chinese-Australian youth. Further investigation into the reliability of the sedentary items within the CAPANS-PA is required before these items can be used with confidence. This study is novel in that the reliability of instruments among CALD groups nationally and internationally remains sparse and this study contributes to the wider body of available psychometrically tested instruments. In addition, this study is the first to our knowledge to successfully engage and investigate the basic health enhancing behaviours of Chinese-Australian adolescents.

## Background

The health benefits of regular physical activity (PA) for children and adolescents are well documented and include a reduced risk of cardiovascular disease, improved insulin resistance, reduction in high blood pressure, improved lipid profile (e.g. lowered high-density lipoprotein cholesterol), and weight management as well as reduced chronic disease-related burden later in life [1-3]. In order to accurately investigate trends, evaluate interventions, establish health relationships, make cross-cultural comparisons, and compare participation to national recommendations [4]; valid and reliable measures of habitual physical activity participation are needed. Without the existence of a 'gold standard' measure that can measure all of the dimensions of physical activity (frequency, intensity, duration, type/mode and domains) the selection of an instrument over another involves a raft of decisions. These decisions surround the key attributes of the instrument and considerations of the population, sample size, respondent burden, method/delivery mode, time frame, data output, data management, psychometric properties, measurement bias and cost of administration [5].

Several subjective (self-report & proxy-report recall questionnaires, diaries/logs and interviewer administered questionnaires) and objective techniques (motion sensors, direct observation, heart rate monitors, doubly labeled water and indirect calorimetry) have been used to measure child and adolescent physical activity levels [6,7]. Although self-report questionnaires have been shown to be prone to misinterpretation, socially desirable responses and overestimation of physical activity in adolescents [8], they remain commonly used at the population level because they are relatively inexpensive and easy to use [9]. Self-report questionnaires can also capture additional information which objective measures cannot provide such as type, domains and correlates of physical activity. Whilst numerous PA activity questionnaires exist for children and adolescents very few have been validated among culturally and linguistically diverse (CALD) youth, a subgroup known to have differing physical activity behaviours both in Australia [10-13] and internationally [14-17]. These differences may partially relate to the ability of the instrument to accurately capture the activities predominantly performed by the CALD group as well as differences in interpretation of PA constructs [18,19]. Therefore there is an urgent need to validate and test the reliability of PA measures for diverse populations, including CALD youth.

Previous research also suggests that variation exists in the reliability and validity of self-report questionnaires among youth [20,21]. However, hampering comparisons of questionnaires between populations is the wide variation in administration techniques (self-completed, interviewer administered, computer assisted), recall period (1-7 days, 1 month - 1 year) [22] and questionnaire wording ('typical week', 'usual week' and 'last week'). A study by Moore et al., 2007 examined the psychometric properties of the Physical Activity Questionnaire for Older Children (PAQ-C) among self-reported Hispanic, African American and European American children (8-11 y) in two studies [21]. Consistent racial variations in construct validity of the PAQ-C score were observed with European American children having stronger correlations with cardiorespiratory fitness, blood pressure and BMI than African American and Hispanic children [21]. Additionally, the internal consistency (Cronbach's α) of the PAQ-C instrument was lower among African American children (α = 0.56) than European American children (α = 0.75) [21]. These alpha levels are considerably lower when compared to the values achieved in the predominantly white Canadian studies among older children (*α *= 0.79-0.89) [23]. These differences in internal consistency of the PAQ-C could theoretically be related to age and age related comprehension abilities [21] as well as different cultural interpretations of physical activity terms. The Canadian studies involved students aged 9-14 y and therefore definitive conclusions regarding the internal consistency are not possible. A more recent study by Huang, Wong and Salmon, 2009 investigated the reliability and validity of the Children's Leisure Activities Study Survey (CLASS), which was originally developed and validated among Australian children (10-12 y) [24], among Hong Kong children (9-12 y) [20]. When comparing the Australian validation study to the Hong Kong study, the 7 day test-retest reliability (Intra Class Correlations, ICC) for time spent in weekly Moderate to Vigorous Physical Activity (MVPA) was higher among Hong Kong children (ICC = 0.71) [20] than Australian children (ICC = 0.24) [24]. Criterion validity of the Hong Kong questionnaire for time spent in MVPA with accelerometry was moderate for girls (ρ = 0.48, p < 0.05) but not for boys (ρ = 0.27, NS)[20] and undesirably low for total activity in the Australian study (ρ = -0.04, NS) [24]. The higher reliability observed in the Hong Kong study may partially be attributed to the differences in the wording of each questionnaire from 'typical week' in the Australian study to 'last week' in the Hong Kong study. This assertion is supported by the literature, with questionnaires involving a shorter period of recall (e.g. 1 to 7 days) tending to generally have higher reliability [6,22,25,26]. Overall, the differences in psychometric properties among youth populations may be resultant of a culmination of factors including, but not limited to the ability of children to accurately recall the duration of physical activity [24], age differences, instrument/study variations and/or cultural differences in the stability of PA over time as well as interpretations of PA constructs (i.e. intensity and duration [18]). The effects of these variables warrant further investigations.

As PA behaviours vary among CALD groups, accurate and reliable measures that are specific to the CALD group are essential. Therefore, the aim of the current study was to investigate the test-retest reliability of a modified version of an instrument previously used in populations of Australian children and adolescents, the Child and Adolescent Physical Activity and Nutrition Survey (CAPANS) questionnaire among Chinese-Australian youth aged 11-14 years. The CAPANS survey involved a battery of both PA and Nutrition questionnaires. This paper will report on the reliability of the PA instrument (CAPANS-PA).

## Methods

### Sampling and recruitment

Convenience samples of three Chinese weekend cultural schools from eastern metropolitan Melbourne were invited to participate in this study. One school declined to participate. The two schools that accepted to participate comprised of three separate school campuses. All students aged 11-16 years were invited to participate (n = 505) through the Plain language statement; with informed consent obtained from the student's parents/guardians and the student themselves. Of the 505 invited students, 106 accepted our invitation and returned their completed questionnaires at baseline, giving a baseline response rate of 21%. Of the 106 students participating at baseline, 29 were absent during post-testing, thus post testing data were obtained on 77 students, giving a follow-up response of 73%. To increase the response rate, repeated visits to each school prior to the planned survey administration day were conducted in order to prompt, remind and encourage students to return their signed consent forms if they wished to participate in the study. This strategy also increased the overall data collection time frame as only one researcher was able to attended each school during these visits, which, due to the nature of Chinese weekend cultural schools only running for 3-4 hours on either a Saturday or Sunday, made attendance at two schools on one day impossible. The final study sample consisted of 77 students, aged 11-14 y at baseline (mean ± SD: 12 ± 0.8 years), with an almost equal distribution between males (n = 38) and females (n = 39). The majority of participants (82%) were of Chinese ethnicity by either being born in China themselves, having both parents born in China or having both maternal grandparents being born in China (Ex. Taiwan, Hong Kong/Macau) at baseline. The study design was approved by the Faculty of Health, Medicine, Nursing and Behavioural Sciences Human Ethics Advisory Group of Deakin University and the Victorian Department of Education and Early Childhood Development.

### Measures

The CAPANS-PA (see Additional file 1) is a 7-day, 20 item recall questionnaire containing several demographic questions and requires participants to indicate their frequency (number of bouts performed) and duration (minutes per bout of participation) among a checklist of 46 commonly performed physical activities and sedentary behaviours (Mon-Fri, Sat & Sun) as well as engagement in school based and non-school based activities (Physical Education, recess and lunch PA, out of school PA) [27]. The questionnaire is almost an exact replica of the Western Australian (WA) Child and Adolescent Physical Activity and Nutrition Survey, Years 8, 10 and 11 questionnaire (CAPANS) which investigates school and non-school based physical activity, sedentary behaviours and associated correlates and its development has been reported elsewhere [27,28]. For the purposes of our study, the singular CAPANS questionnaire was separated into two distinct questionnaires with the first (CAPANS-PA) questionnaire investigating physical activity and sedentary behaviours and the second (CAPANS-BA) investigating self-reported barriers to physical activity participation. Both instruments were utilised with permission from its original developer the Evaluation and Monitoring Working Group of the Western Australian Premier's Physical Activity Taskforce (PATF).

The items within the CAPANS-PA questionnaire derive from several sources including the Children's Leisure Activities Study (CLASS) Survey [24], the Physical Activity Questionnaire for Adolescents [29], PAQ-C [23] the APARQ [30], the 1985 Australian Health and Fitness Survey (AHFS)[31] and the Self-Administered Activity Checklist [32] (see Additional file 2). After the development of the CAPANS questionnaire was completed, the reliability of the instrument was investigated among 65 Grade 3 students in WA whom completed the questionnaire on two occasions, 7-days apart. Agreement between individual items ranged from low to high (k 0.10-0.83) [27].

Modifications made to the CAPANS-PA instrument from the original were minor and included the addition of country of birth of the participant, parents and maternal grandparents, rewording from 'usually do during a typical week' to 'last 7 days' as reliability increases with a shorter period of recall [6,22,25,26]. This change to 'last 7 days' was also adopted in the 2008 CAPANS WA study from the original 2003 version [33]. Additionally, the '4 square/Downball' item was removed and replaced with 'hockey'(to reflect field hockey), 'surfing' and 'martial arts' as recommended by the PATF in their 2003 report [34]. Changes to the question surrounding sedentary behaviours included the creation of four items from the original two items 'Study, homework or extra tutoring' and 'Go to church or Saturday school' into 'Study or do homework', 'Attend Saturday school', 'Go to church' and 'Attend out-of-school hours tutoring'. No changes were made to the 6 school based and non-school based PA questions which requires participants to report their frequency of activity after school, in the evenings and last weekend on a 5-point likert scale as well as rate how active they were in physical education, recess and lunch over the past 7 days using a 5-point likert scale.

### Procedure

Data collection occurred between May and August 2009. Students' completed the self-report questionnaire at baseline and 7-days later during class-time, which took approximately 15 minutes to complete on each occasion. The selection of a 7-day test-retest period was guided by the recommendations by Sallis & Saelens (2000) which indicate that the test-retest interval of one week self-report recalls be repeated within one week, as with one day recalls be repeated on the same day [35]. Participants were guided through the questionnaire by the investigator or trained data collectors. To ensure all instructions and responses to questions were consistent, data collectors attended a 1 hour training session led by the investigator and were issued a training manual. This manual contained the standardised questionnaire administration procedure and a list of standardised responses to potential questions. At the end of the second questionnaire administration, each student received compensation in the form of a recyclable bag containing a 'hacky sack' and three Australian Government brochures pertaining to healthy eating and regular physical activity for children and adolescents.

The implementation of the research was seen by a research advisory community composed of experts in PA and nutrition as well as a member of the Chinese associations. The role of the advisory committee was to comment of the instruments, advise on appropriate recruitment methods, comment of the research findings, and to promote a co-learning and empowering process in order to attend to health inequalities in among CALD communities [36].

### Data management and analysis

The completed questionnaires were hand screened for errors and entered into SPSS Statistics Version 17.0 for analysis. Ten percent of the questionnaires (n = 8) were randomly selected and re-entered for both baseline and post-testing questionnaires for quality assurance. Reported participation in the 32 PA activities (e.g. Frequency Aerobics Mon- Fri + Frequency Dance Mon - Fri and so forth) was summed up to generate four PA totals for frequency of participation: Total PA Frequency Mon-Fri; Total PA frequency Sat; Total PA frequency Sun; and Total PA frequency Mon-Fri, Sat and Sun. This process was repeated for duration to create the four variables (Total PA Duration Mon-Fri, Total PA Duration Sat, Total PA Duration Sun and Total PA Duration Mon-Fri, Sat and Sun). The results were also examined for extreme values where reported duration of participation exceeded 6 hours/day, this was an arbitrary cut point. Therefore, the responses of participants who reported participation (> 1800 min/Mon-Fri) or (> 360 min/Sat or Sun) were excluded from the analysis for the particular question only, resulting in data being excluded from five participants. Activities from the 32-item list of common physical activities were assigned MET values based on moderate intensity effort from the compendium of energy expenditures for youth [37]; in order to investigate the reliability of the instrument in estimating frequency and duration of participation in MVPA activities.

Five categories of sedentary behaviours were created from the 14 item checklist based on those developed by Hardy et al., 2007 [38]. The results were examined for extreme values where reported duration of participation exceeded 8 hours/day Mon- Fri and 16 hours/day Sat or Sun, as the questionnaire related to out of school hours sedentary behaviours, this was an arbitrary cut point. Therefore, the responses of participants who reported sedentary behaviours (> 2400 min/Mon-Fri) or (> 960 min/Sat or Sun) were excluded from the analysis, resulting in data being excluded from 12 participants for this question (16%).

### Reliability of the CAPANS-PA Questionnaire

Initial analyses conducted were paired-sample t-tests for parametric variables and McNemar's test for non-parametric variables to determine whether there was a significant group level difference (p < 0.05) between baseline and post-testing means on all individual survey items, except for sedentary activities, where the activity total was used [39]. The McNemar test is a direct equivalent of the paired t-test for parametric variables and indicates whether or not reported group-level participation in an activity was significantly different from baseline to post-testing [40]. This is different to reliability which examines whether an individuals' reported participation differed significantly from test to retest.

Test-retest reliability of the CAPANS-PA questionnaire, was assessed by ICC's (one-way random model) for parametric (interval and ratio) variables with acceptable reliability defined as an ICC ≥ 0.70 [41]. For non-parametric (nominal and ordinal) variables, the Cohen's Kappa statistics (*k*) was used to examine reliability with poor agreement set at 0%, slight agreement (0.00-0.20), fair agreement (0.21-0.40), moderate agreement (0.41-0.60), substantial agreement (0.61-0.80) and almost perfect agreement (0.81-1.00) [42].

## Results

### Test-retest reliability of individual PA items

Reliability of participation (Yes/No) among the list of 32 individual physical activities demonstrated the majority of items had at least substantial agreement with (4 items) having almost perfect, (19 items) substantial and (7 items) moderate agreement with (2 items) having negative agreements (Table 1). The McNemar tests also revealed no significant differences in reported participation (Yes/No) for most of the physical activities, except for tennis and skipping rope activities.

**Table 1:** Difference in means and test-retest reliability of individual PA items

*Activity*	*MET^a ^Value & MPA^b ^or VPA^c^*	*Paired t-test (P)*	*Kappa (95% CI)*	*(P)*	*N and % of missing responses/77#*
Aerobics	(6.2)- VPA	1.00	.65 (.39-1.00)	< 0.001	17, 22%
Dance	(5.5)- MPA	1.00	.56 (.27-.84)	< 0.001	14,18%
Callisthenics/ Gymnastics	(5.8)- MPA (4.0)- MPA	1.00	.61 (.29-.94)	< 0.001	16, 20%
Tennis/Table tennis	(7.0)- VPA (4.0)- MPA	.02*	.71 (.51-.87)	< 0.001	9, 12%
Australian Rules Football	(8.8)- VPA	.58	.50 (.27-.73)	< 0.001	19, 25%
Soccer	(8.8)- VPA	.06	.56 (.36-.76)	< 0.001	13,17%
Basketball	(8.2)- VPA	.18	.71 (.53-.88)	< 0.001	16, 21%
Cricket	(3.5)- MPA	.69	.52 (.18-.85)	< 0.001	17, 21%
Netball	(8.2)- VPA	.45	.70 (.05-.91)	< 0.001	14,18%
Baseball/Softball	(5.0)- MPA (5.0)- MPA	1.00	.68 (.15-.98)	< 0.001	15, 20%
4 square/Down ball	(5.0)- VPA	.75	.69 (.50-.86)	< 0.001	11,14%
Swimming laps	(9.9)- VPA	.18	.71 (.54-.88)	< 0.001	14,18%
Tag/chasey	(5.0)- MPA	.77	.62 (.43-.81)	< 0.001	11,14%
Skipping rope	(8.3)- VPA	.04*	.58 (.35-.82)	< 0.001	14,18%
Martial Arts	(10.0)-VPA	1.00	1.0 (n/a)	< 0.001	16, 21%
Hockey	(8.0)- VPA	1.00	.74 (.36-1.00)	< 0.001	19, 25%
Surfing	(5.0)- MPA	1.00	.66 (.04-1.00)	< 0.001	20, 26%
Roller-blading	(6.5)- VPA	1.00	.64 (.41-1.00)	< 0.001	18, 23%
Scooter	(6.5)- VPA	1.00	.64 (.30-.94)	< 0.001	15, 20%
Skateboarding	(5.0)- MPA	1.00	1.0 (n/a)	< 0.001	18, 23%
Bike riding	(6.2)- VPA	.07	.60 (.39-.80)	< 0.001	15, 20%
Household chores		.12	.38 (.11-.59)	< 0.001	8, 10%
Play on playground equipment	(5.0)- MPA	.07	.68 (.48-.88)	< 0.001	14,18%
Play in cubby house	(5.0)- MPA	1.00	-.02 (-.05-.01)	.86	17, 22%
Bounce on trampoline	(8.7)- VPA	.51	.39 (.07-.71)	0.002	15, 20%
Play with pets	(4.0)- MPA	.63	.79 (.60-.99)	< 0.001	15, 20%
Walk the dog	(4.0)- MPA	.50	.82 (.57-1.00)	< 0.001	16, 21%
Walk for exercise	(3.6)- MPA	.75	.61 (.36-.79)	< 0.001	11,14%
Jogging or running	(8.5)- VPA	.18	.56 (.33-.76)	< 0.001	9, 12%
Physical and Sport Education class		.69	-0.04 (-0.07-0)	.73	3, 4%
Travel by walking to school carrying load e.g. School bag	(4.2)- MPA	1.00	.75 (.59-.93)	< 0.001	13,17%
Travel by cycling to school	(6.2)- VPA	1.00	.79 (.40-1.00)	< 0.001	17, 22%
Other (Please state)		.38	.65 (.29-.92)	< 0.001	47, 61%
Other (Please state)		1.000	1.0 (n/a)	< 0.001	58, 75%

* p < 0.05; N = sample size; # = The total number and percentage of missing responses between test and retest from 77 participants; n/a = results not applicable; ^a = ^equivalent metabolic value based on moderate intensity effort; ^b ^= moderate intensity physical activity; ^c ^= vigorous intensity physical activity

### Frequency and duration of MVPA

Frequency of MVPA participation per day (Mon-Fri, Sat & Sun) at baseline was on average 3 bouts/day (mean ± SD: 3 ± 2.5 bouts) with duration being 98 mins/day (mean ± SD: 98 ± 52 mins), with no significant difference occurring between baseline and post-testing for this total. Gender differences were also evident for frequency and duration of MVPA participation (Mon-Fri, Sat & Sun) at baseline with males having higher a frequency (males mean ± SD: 24.8 ± 14.2; females mean ± SD: 22.1 ± 20.5) and duration of MVPA (males mean ± SD: 791.9 ± 367.5; females mean ± SD: 580.0 ± 332.4) than females. Tables 2, 3, 4 and 5 display the test-retest reliability results and paired t-tests for the frequency and duration totals of MVPA by gender. Acceptable reliability (ICC≥0.70) was observed for total physical activity frequency (Mon-Fri), (Mon-Fri, Sat & Sun) and Saturday for females, with males not achieving acceptable reliability for frequency of MVPA. When examining the reliability of time spent in MVPA, males had acceptable reliability for duration of total MVPA (Mon-Fri) and (Mon-Fri, Sat & Sun) participation, whilst females had acceptable reliability for total MVPA duration (Mon-Fri), (Mon-Fri, Sat & Sun) and Saturday.

**Table 2:** Gender differences in test-retest reliability for frequency MVPA totals for Mon-Fri and Saturday

*Total MVPA^b ^Monday- Friday*						*Total MVPA^b ^Saturday*				
	**N**	**Baseline bouts^d^(SD)**	**Post-testing bouts^d^(SD)**	**Difference^c ^D(95%: CI)**	**ICC^a ^(95%: CI)**	**N**	**Baseline bouts^d ^(SD)**	**Post-testing bouts^d ^(SD)**	**Difference^c ^D(95%: CI)**	**ICC^a ^(95%: CI)**

All	76	19.6 (11.5)	18.0 (10.5)	1.6 (-.06-3.37)	.77** (.67-.85)	47	3.06 (4.7)	4.0 (6.0)	-0.9 (-2.1-.22)	.73** (.57-.84)
Male	37	21.4 (11.9)*	18.1 (8.9)	3.3 (.47-6.01)	65** (.42-.80)	23	2.5 (2.1)	3.5 (5.3)	-1.0 (-3.2-1.22)	.18 (-.23-.55)
Female	39	17.9 (11.1)	17.9 (12.0)	0.0 (-1.86-1.96)	.87** (.77-.93)	24	3.6 (6.3)	4.5 (6.7)	-0.9 (-1.77-.10)	.94** (.86-.97)

^a ^Intra-class correlation and 95% confidence interval; ^b ^= moderate to vigorous physical activity participation; *p < .05, **p < 0.01, N = number of respondents for the question item; ^c ^mean difference from baseline to post-testing using paired sample t-tests; ^d ^mean bouts and significance

**Table 3:** Gender differences in test-retest reliability for frequency MVPA totals for Sunday and Mon-Fri, Sat & Sun

*Total MVPA^b ^Sunday*						*Total MVPA^b ^Mon-Fri, Sat & Sun*				
	**N**	**Baseline^d ^bouts (SD)**	**Post-testing bouts^d ^(SD)**	**Difference^c ^D(95%: CI)**	**ICC^a ^(95%: CI)**	**N**	**Baseline bouts^d^(SD)**	**Post-testing bouts^d ^(SD)**	**Difference^c ^D(95%: CI)**	**ICC^a ^(95%: CI)**

All	32	2.8 (7.2)	2.4 (1.7)	0.4 (-2.00-2.81)	.19 (-.16-.50)	76	23.4 (17.7)*	21.5 (15.3)	1.9 (.01-3.91)	.86**(.79-.91)
Male	15	1.4 (1.9)	2.2 (1.6)	-0.8 (-1.87-.27)	.35 (-.16-.72)	37	24.8 (14.2)*	21.2 (11.9)	3.6 (.08-7.11)	.65** .42-.81)
Female	17	4.0 (9.6)	2.5 (1.8)	1.5 (-3.14-6.08)	.18 (-.31-.59)	39	22.1 (20.5)	21.7 (18.0)	0.4 (-1.48-2.30)	.96** (.92-.98)

^a ^Intra-class correlation and 95% confidence interval; ^b ^= moderate to vigorous physical activity participation; *p < .05, **p < 0.01, N = number of respondents for the question item; ^c ^mean difference from baseline to post-testing using paired sample t-tests; ^d ^mean bouts and significance

**Table 4:** Gender differences in test-retest reliability for duration MVPA totals for Mon-Fri and Saturday

*Total MVPA^b ^Monday- Friday*						*Total MVPA^b ^Saturday*				
	**N**	**Baseline Mins^d ^(SD)**	**Post-testing Mins^d ^(SD)**	**Difference^c ^D(95%: CI)**	**ICC^a ^(95%: CI)**	**N**	**Baseline Mins^d ^(SD)**	**Post-testing Mins^d ^(SD)**	**Difference^c ^D(95%: CI)**	**ICC^a ^(95%: CI)**

All	72	582.6 (299.8)	554.0 (309.5)	28.6 (-22.79-79.98)	.74** (.62-.83)	44	84.7(81.9)*	107.5 (88.3)	-22.8 (-42.04--3.46)	.70** (.51-.82)
Male	37	669.7 (303.0)	613.3 (289.3)	56.4 (-11.08-123.89)	.76** (.58-.87)	23	82.6 (72.1)	104.4 (91.7)	-21.7 (-49.95- 6.47)	.67** (.37-.84)
Female	35	490.5 (271.1)	491.2 (321.8)	-0.8 (-81.18-79.58)	.70** (.48-.84)	21	87.1 (93.3)	110.9 (86.5)	-23.9 (-52.56-4.84)	.73** (.46-.88)

^a ^Intra-class correlation and 95% confidence interval; ^b ^= moderate to vigorous physical activity participation; *p < .05, **p < 0.01, N = number of respondents for the question item; ^c ^mean difference and significance from baseline to post-testing using paired sample t-tests; ^d ^mean minutes spent in MVPA

**Table 5:** Gender differences in test-retest reliability for duration MVPA totals for Sunday and Mon-Fri, Sat & Sun

*Total MVPA^b ^Sunday*							*Total MVPA^b ^Mon-Fri, Sat & Sun*			
	**N**	**Baseline Mins^d ^(SD)**	**Post-testing Mins^d ^(SD)**	**Difference^c ^D(95%: CI)**	**ICC^a ^(95%: CI)**	**N**	**Baseline Mins^d ^(SD)**	**Post-testing Mins^d ^(SD)**	**Difference^c ^D(95%: CI)**	**ICC^a ^(95%: CI)**

All	30	65.6 (80.4)	88.3 (64.0)	-22.7 (-52.94-7.47)	.36* (.01-.63)	72	688.8 (364.4)	656.5 (364.4)	32.4 (-24.96-89.71)	.78** (.66-.85)
Male	15	54.0 (70.3)	94.0 (65.0)	-40.0 (-82.76-2.76)	.27 (-.25-.67)	37	791.9 (367.5)	716.3 (338.0)	75.60 (-10.88-162.07)	.72** (.52-.84)
Female	15	77.2 (90.4)	82.7 (64.6)	-5.5 (-51.64-40.71)	.46* (-.03-.78)	35	580.0 (332.4)	593.3 (385.1)	-13.3 (-89.29-62.66)	.82** (.67-.90)

^a ^Intra-class correlation and 95% confidence interval; ^b ^= moderate to vigorous physical activity participation; *p < .05, **p < 0.01, N = number of respondents for the question item; ^c ^mean difference and significance from baseline to post-testing using paired sample t-tests; ^d ^mean minutes spent in MVPA

Despite these gender differences the CAPANS-PA questionnaire provided reliable estimates of frequency and duration of total MVPA participation (Mon-Fri), (Mon-Fri, Sat & Sun) and Saturday for all participants.

### School based and non-school based PA

The reliability of self-reported activity in a variety of school based and non-school based PA is presented in Table 6. There were no significant differences in mean responses from baseline to post-testing for all participants across any of the school based and non-school based activities, with the only differences observed for female activities at lunch and recess, and male activities at recess (p < 0.05). Overall, all items had at least fair agreement for all participants, males and females with frequency of physical activity participation in the evenings (6-9 pm) and very active participation in Physical Education being the most stable items for all participants. In general, the least stable items were activities conducted at lunch for all participants and females, with frequency of physical activity participation in the evenings (6-9 pm) being the least stable for males.

**Table 6:** Test-retest reliability of school based and non-school based activity questions

	ALL		Males		Females	
	
	N: (McNemar test p-value^a ^	Kappa^b ^(95%: CI)	N: (McNemar test p-value^a ^	Kappa^b ^(95%: CI)	N: (McNemar test p-value^a^	Kappa^b ^(95%: CI)
Frequency of very active participation in physical education	72 (.24)	.51 (.34, .67)	37 (.20)	.47 (.20, .69)	36 (.77)	.52 (.29, .76)
Activity at recess (besides eating)	77 (.90)	.49 (.35, .63)	38 (.00**)	.53 (.33, .73)	39 (.00**)	.42 (.20, .64)
Activity at lunch (besides eating)	77 (.13)	.34 (.19, .48)	38 (.86)	.37 (.17, .58)	39 (.04*)	.29 (.08, .49)
Frequency of physical activity engagement right after school (3:30-6 pm)	77 (.78)	.48 (.37, .66)	38 (.30)	.43 (.21, .66)	39 (.38)	.52 (.32, .72)
Frequency of physical activity engagement in the evenings (6-9 pm)	77 (.78)	.50 (.37, .66)	38 (.49)	.33 (.11, .56)	39 (.64)	.64 (.45, .83)
Frequency of physical activity engagement during the last weekend	77 (.49)	.49 (.34, .64)	38 (.28)	.53 (.30, .75)	39 (1.00)	.43 (.22, .65)

*p < .05; ^a ^= McNemar test to assess significant differences in response means from baseline and post-testing; N = number of respondents for the question item; ^b ^= Cohen's Kappa statistic with 95% confidence interval

### Sedentary behaviours

Table 7 displays the reliability results and paired t-tests for duration spent in various sedentary activities by gender based on the categories of sedentary behaviours developed by Hardy et al., 2007(Small Screen Recreation, Education, Cultural, Social and Travel) [37]. When contrasting engagement in weekly sedentary activities (Mon-Fri, Sat & Sun) females had higher reliability coefficients than males and the total population (Females ICC = 0.83, p < 0.01; Males ICC = 0.42, p < 0.01; All ICC = 0. 64, p < 0.01). In general, across activity categories, the reliability of weekday behaviours (Mon-Fri) tended to be higher than weekend behaviours (Sat or Sun) for all participants, with the duration of Small Screen Recreation (ICC = 0.72, p < 0.01) and Cultural activities (ICC = 0.75, p < 0.01) being the most reliable behaviour for all participants (Mon-Fri). The least reliable behaviour for all participants, males and females was engagement in social activities (Mon-Fri), however, the reliability of this behaviour increased markedly for Saturday and Sunday (Table 7.). However, it should be noted that reported participation in this activity was low for both genders and thus, these findings should be interpreted with caution.

**Table 7:** Test-retest reliability and paired-sample t-tests for duration in sedentary activity

	*Monday-Friday*		*Saturday*		*Sunday*	
	T-test^c^: **N (p-value)**	ICC^a ^(95%: CI)	T-test^c^: **N (p-value)**	ICC^a ^(95%: CI)	T-test^c^: **N (p-value)**	ICC^a ^(95%: CI)
**Total Small Screen Recreation**						
**All**	65 (NS)	.72** (.58-.82)	65 (NS)	.52** (.32-.68)	65 (NS)	.58** (.39-.72)
**Male**	34 (NS)	.53*** (.24-.73)	34 (NS)	.52** (.23-.73)	34 (NS)	.44** (.13-.67)
**Female**	31 (NS)	.86*** (.73-.93)	31 (NS)	.50** (.19-.72)	31 (NS)	.79*** (.60-.89)
**Total Education**						
**All**	65 (NS)	.59** (.41-.73)	65 (NS)	.62** (.44-.75)	65*	.65** (.49-.77)
**Male**	33 (NS)	.64*** (.39-.80)	34 (NS)	.37* (.04-.63)	34 (NS)	.72*** (.51-.85)
**Female**	31 (NS)	.47** (.15-.71)	31 (NS)	.83*** (.68-.91)	31 (NS)	.55 ** (.25-.75)
**Total Travel**						
**All**	37 (NS)	.67** (.47-.82)	29 (NS)	.29 (-.08-.59)	27 (NS)	.47* (.12-.72)
**Male**	17 (NS)	.55** (.12-.81)	14 (NS)	.18 (-.36-.63)	11 (NS)	.59* (.05-.87)
**Female**	20 (NS)	.82*** (.61-.93)	15 (NS)	.33 (-.18-.71)	16 (NS)	.45 * (-.03-.76)
**Total Cultural Activities**						
**All**	65 (NS)	.75** (.62-.84)	65 (NS)	.62** (.45-.75)	61 (NS)	.64** (.48-.77)
**Male**	34 (NS)	.65*** (.40-.81)	34 (NS)	.59*** (.32-.77)	34 (NS)	.64*** (.39-.80)
**Female**	31 (NS)	.86*** (.74-.93)	31 (NS)	.65*** (.39-.81)	31 (NS)	.65*** (.39-.81)
**Total Social Activities**						
**All**	65 (NS)	.03 (-.21-.27)	65 (NS)	.43** (.21-.61)	65 (NS)	.75** (.61-.84)
**Male**	34 (NS)	.02 (-.31-.35)	34 (NS)	.36* (.03-.62)	34 (NS)	.68 ***(.45-.83)
**Female**	31 (NS)	.09 (-.27-.42)	31 (NS)	.49** (.17-.71)	31 (NS)	.79 ***(.61-.89)
**All SEDENTARY ACTIVITIES**						
**All**	65 (NS)	.43** (.21-.61)	65 (NS)	.57** (.38-.71)	65**	.65** (.48-.77)
**Male**	34 (NS)	.41** (.09-.66)	34*	.47** (.17-.70)	.34**	.52** (.23-.73)
**Female**	31 (NS)	.81** (.64-.90)	31 (NS)	.67*** (.43-.83)	31 (NS)	.79*** (.61-.89)

^a ^Intra-class correlation; *p < .05; **p < 0.01; n = number of respondents for the question item

## Discussion

This study was the first to our knowledge to examine the reliability of a questionnaire investigating physical and sedentary behaviours among Chinese-Australian youths. The results suggest that the instrument provides reliable estimates of type, frequency and duration of weekly MVPA participation for all participants. Time spent in weekly sedentary behaviours (Mon-Fri, Sat & Sun) were reliable for females only, with males only having acceptable reliability on certain days and within specific activity categories (e.g. small screen recreation, total travel, and cultural activities), rather than overall behaviours. The reliability of the questionnaire appears to be comparable with existing instruments (other than the CAPANS-PA) that assess weekly (Mon-Sat) time in MVPA or associated PAEE ranging from ICC = 0.24 - 0.98 [22,24,43] and weekly time spent in sedentary activities ranging from ICC = 0 .57 -0.86 [38,43] among youths.

The test-retest reliability of type, frequency and duration of PA and sedentary activities conducted in the previous week was reported using the CAPANS-PA questionnaire and examined on two occasions, one week apart. Engagement in MVPA had at least moderate agreement for the majority of individual PA checklist items, with frequency and duration of participation varying widely in reliability. The two negative agreements observed for participation (Yes/No) in 'Physical and Sport Education class at school and Play in cubby house' are abnormal however, can occur and relates to the phrasing of the particular question, as those students who answered 'yes' or 'no' to participation in 'physical and sport education' and 'playing in a cubby house' are unlikely to change their response between testing (e.g. acquire a cubby house to play in between testing). In general, the ICC's for MVPA for all participants were much higher for Mon-Fri than Saturday or Sunday for both frequency and duration. This suggests that participants had difficulty in accurately recalling weekend activities, especially on Sunday, as no significant differences in frequency or duration of MVPA participation were observed for Sunday participation. Gender differences in reliability were also evident with female ICCs for frequency and duration of MVPA participation (Mon-Fri, Sat and Sun) being higher than males. This theoretically may relate to females engaging in less physical activity than males overall [44], and thus needing to recall less activities. This finding is similar to the 3-Day Physical Activity Recall (3DPAR) validation study among Singaporean adolescents, where males had higher ICC's for weekday (Mon and Tue) than Weekend (Sun) activities, although these were not higher than female ICCs [45].

Our results are similar to those observed in the CLASS [20,24] validation studies, from which these questionnaire items were heavily based. Similar to the Hong Kong study our ICC's for duration in MVPA Mon-Fri, Sat and Sun were higher than the Australian study, which may partly be explained by the older age of the participants in this study, as age is associated with the recall and cognitive ability of participants [46] and higher ICC's have been observed for older (16-18 y) compared to younger adolescents (13-15 y) [47] as well as the change in wording of the questionnaire from 'usual activity' in the Australian study to 'last week' in the Hong Kong and present study. In addition, it is possible that PA behaviours of Chinese-Australian youths may be similar to their Hong Kong counterparts in both stability of PA behaviour, conceptualisations of PA constructs and engagement in similar activities. Further studies are required to confirm this assertion.

The reliability results of school based and non-school based PA were generally similar across genders, except for engagement in activities at recess and lunch and weekend activities, which were less stable for females. However, intra-individual variation was evident for recess and lunch activity of females, from baseline to post-testing, which theoretically may account for the observed differences in reliability. These question items predominantly derive from the Physical Activity Questionnaire for Adolescents [29] and the PAQ-C [23] questionnaire. Comparisons of our test-retest reliability results with the previous validation studies are not possible, because of differences in how the questionnaires were interpreted. The PAQ-C questionnaire contains 9 items, including an activity checklist, which is subsequently used to generate a PAQ-C score ranging from 1 to 5 which reflect total activity [23]. The test-retest reliability of the entire PAQ-C questionnaire is reported as a single score and school based and non-school based PA item are not separated [26]. Our results indicate these questionnaire items had fair to substantial agreement and thus are deemed reliable.

The reliability for all sedentary activities (Mon-Fri, Sat & Sun) was only acceptable for females (ICC = 0.83). However, when these sedentary behaviours were split into like categories based on the ASAQ [38], the ICC's of related activities were much stronger. The social activities category was not reliable for Mon- Fri or Saturday for any participant, regardless of gender; thus warranting further investigation as no significant intra-individual difference in duration of engagement were observed from baseline to post testing. There are two possible reasons for the observed low ICCs and include poor reliability in the participant reporting participation or the behaviour is not stable over time and experiences intra-individual variability. In this instance, it would appear that the reliability of reporting participation in social activities is low, especially Mon-Fri. When comparing our reliability results to the ASAQ, the Dutch Activity Questionnaire for Adults and Adolescents (AQuAA) [43] and the Hong Kong CLASS validations, the results for time spent in weekly sedentary activities are comparable, with no questionnaire having acceptable reliability for all age groups. Total duration of sedentary activities (Mon-Fri, Sat & Sun) ranged from ICC = 0.57- 0.86 in the ASAQ [38] (11-15 y), ICC = 0.69 in Hong Kong CLASS [20] (9-12) years, ICC = 0.57 in AQuAA (13-15 y) [43] with our results having an ICC = 0.64. Caution needs to be taken when interpreting our results as 16% of the original respondents were excluded due to over-reporting sedentary participation, beyond what was considered 'achievable' during a normal school week or weekend. However, when these results were included in the analysis, the reliability coefficients were surprisingly higher, suggesting that over-reporters were consistently over-reporting their sedentary activity.

An important characteristic of this study was the successful engagement and recruitment of Chinese-Australian adolescents through Chinese weekend cultural schools, with an even ratio of males to female participants. To our knowledge, this is the first study to investigate basic health enhancing behaviours among the adolescent Chinese-Australian community. Additionally, the CAPANS-PA questionnaire provides a relatively quick, easy to complete and cost effective technique to examine the physical and sedentary behaviours of adolescents', which is ideal for large scale studies. It is likely that the CAPANS-PA questionnaire would provide similarly reliable estimates of adolescent physical activity levels among other 'Asian' (e.g. Vietnamese, Cambodian, Malaysian, Korean, Thai etc) populations in English speaking countries. However, several limitations should be considered when interpreting the findings from this study. Firstly, the use of a convenience sample restricts the generalisability of our results to the wider population, however this was deemed necessary in order to recruit sufficient numbers of the target population. Secondly the small sample size and low response rate introduces large selection bias into the results, especially in the un-powered gender analyses, and may over or understate the results achieved when used in the wider population. The low response rate observed in this study was not entirely unexpected, as low response rates among CALD/minority groups in health-related research is widely reported [48-52]; in addition to a global downturn in rates of participation in epidemiological studies [48]. It is also of note that the reporting of response rates in physical activity questionnaire validation studies among children and adolescents is less apparent in the literature and should be encouraged with several questionnaires, Hong Kong CLASS, CLASS, AQuAA, PAQ-C, 3DPAR and ASAQ failing to report participant response rates, making comparisons difficult [20,24,43,23,45,38]. Although the low response rate in this study may influence the generalisability of this study, the authors believe the results observed in this study would be reflective of the wider Chinese-Australian adolescent community.

As this study did not assess the validity of the CAPANS-PA questionnaire, reliability alone is an important property of self-report physical activity questionnaires and has been shown to vary among CALD children [20,21]. The mechanisms through which reliability is influenced among CALD populations is not well understood and warrants further investigation, however, our results confirm the need to examine the reliability properties of self-report questionnaires among differing CALD populations. It is also important to provide reliability coefficients for several physical activity behaviours, type (e.g. soccer, dance, aerobics) and duration of specific sedentary activities (e.g. music practice, reading, homework), where validity is not able to be practically assessed. However, it is recommended that future studies using the CAPANS-PA questionnaire involve larger, representative samples of Chinese-Australians and employ the use of an objective criterion measure, such as an accelerometer, to validate duration, frequency and intensity of physical activity. Despite objective measures of physical activity having their own limitations such as cost, time, participant burden and limitations in the number of dimensions of physical activity captured, their employment in validating the questionnaire would be highly advantageous.

## 3.6 Conclusion

Overall, the results suggest that the CAPANS-PA questionnaire is generally reliable for assessing type, frequency and duration of MVPA participation Mon-Fri, Saturday and Sunday for Chinese-Australian adolescents; with sedentary behaviours being reliable for females only (Mon-Fri, Sat and Sun) and total education Sunday for males. This study is novel in that the reliability of instruments among CALD groups nationally and internationally remains sparse, and are still needed. The CAPANS-PA instrument helps add to wider body of available instruments among adolescents, especially those from CALD backgrounds.

## Competing interests

The authors declare that they have no competing interests.

## Authors' contributions

CS directed all aspects of the study, including co-refinement of the CAPANS-PA questionnaire, design of the study, data collection, administration of the questionnaire, data entry and analysis. CS led the writing of the manuscript. CB, AR and KR were involved in the refinement of the CAPANS-PA questionnaire, conception and design of the study, analysis framework and assisted with the critiquing of the manuscript. AR also provided extensive support and practical guidance through the analysis phase of this study. Additionally, all authors have read and approved the final manuscript.

## Pre-publication history

The pre-publication history for this paper can be accessed here:

http://www.biomedcentral.com/1471-2288/11/122/prepub

## Supplementary Material

Additional file 1 **The Modified CAPANS-PA (Physical Activity) Questionnaire**. This file contains a copy of the modified CAPANS-PA questionnaire that was used in this study. It has been provided with permission from the authors of the modified instrument (CAPANS-PA) in addition to the Western Australian Premier's Physical Activity Taskforce (PATF) Secretariat. Click here for file

Additional file 2 **Summary of the CAPANS-PA Questionnaire**. This file contains a summary of the items contained within the CAPANS-PA questionnaire. The summary outlines the original source of the question item, modifications made to the original CAPANS Year 8, 10 and 11 questionnaire and reliability and validity properties where available. Click here for file
