# Supplementary material for: Reliability of the modified child and adolescent physical activity and nutrition survey, physical activity (CAPANS-PA) questionnaire among chinese-australian youth
Source: BMC Med Res Methodol. 2011 Aug 25;11:122. doi: 10.1186/1471-2288-11-122 (PMC3175205; doi:10.1186/1471-2288-11-122)
Supplement: Additional file 1 — The Modified CAPANS-PA (Physical Activity) Questionnaire. This file contains a copy of the modified CAPANS-PA questionnaire that was used in this study. It has been provided with permission from the authors of the modified instrument (CAPANS-PA) in addition to the Western Australian Premier's Physical Activity Taskforce (PATF) Secretariat. [file 1471-2288-11-122-S1.DOC]

The Modified CAPANS-PA (Physical Activity)

Questionnaire

**CAPANS-PA (Child and Adolescent Physical Activity and Nutrition Survey, Physical Activity Questionnaire**

**Name:_____________________________________ Date of Birth: ______________ Age: __________years**

**School:**_________________________________­­­___ **Gender (Circle): Male or Female**

**Which Suburb do you live in: ______________________________________ Postcode:___________**

**The following questions relate to you and your biological family (persons who gave birth to you) (Circle)**

| **1. Which country were you born in?** | **1. Australia**  **2. China (Ex. Taiwan, Hong Kong/ Macau)**  **3. Taiwan, Hong Kong and Macau**  **4. India**  **5. America** | **6. New Zealand**  **7. Canada**  **8. England**  **9. Malaysia**  **10. Vietnam**  **11. Other: ________________________** |
| --- | --- | --- |
| **2. Which country was your mother born in?** | **1. Australia**  **2. China (Ex. Taiwan, Hong Kong/ Macau)**  **3. Taiwan, Hong Kong and Macau**  **4. India**  **5. America** | **6. New Zealand**  **7. Canada**  **8. England**  **9. Malaysia**  **10. Vietnam**  **11. Other: ________________________** |
| **3. Which country was your father born in?** | **1. Australia**  **2. China (Ex. Taiwan, Hong Kong/ Macau)**  **3. Taiwan, Hong Kong and Macau**  **4. India**  **5. America** | **6. New Zealand**  **7. Canada**  **8. England**  **9. Malaysia**  **10. Vietnam**  **11. Other: ________________________** |
| **4. Which country was your grandmother born in?** | **1. Australia**  **2. China (Ex. Taiwan, Hong Kong/ Macau)**  **3. Taiwan, Hong Kong and Macau**  **4. India**  **5. America** | **6. New Zealand**  **7. Canada**  **8. England**  **9. Malaysia**  **10. Vietnam**  **11. Other: ________________________** |
| **5. Which country was your grandfather born in?** | **1. Australia**  **2. China (Ex. Taiwan, Hong Kong/ Macau)**  **3. Taiwan, Hong Kong and Macau**  **4. India**  **5. America** | **6. New Zealand**  **7. Canada**  **8. England**  **9. Malaysia**  **10. Vietnam**  **11. Other: ________________________** |

1. **Which of the following Physical activities did you engage in during the last 7 (seven) days?**

|  |  | **Monday - Friday** | |  | **Saturday** | |  | | **Sunday** | |  |
| --- | --- | --- | --- | --- | --- | --- | --- | --- | --- | --- | --- |
| In the last week which activities did you do? | Did you do this activity? | Number of times | Total Hours/ Minutes | | Number of times | Total Hours/ Minutes | | Number of times | | Total Hours/ Minutes | |
| **E.g. Bike riding** | No_1_ **Yes_2_** | **2** | **40mins** | | **1** | **15mins** | | **1** | | **15mins** | |
| Aerobics | No_1_ Yes_2_ |  |  | |  |  | |  | |  | |
| Dance | No_1_ Yes_2_ |  |  | |  |  | |  | |  | |
| Callisthenics/ gymnastics | No_1_ Yes_2_ |  |  | |  |  | |  | |  | |
| Tennis/Table tennis | No_1_ Yes_2_ |  |  | |  |  | |  | |  | |
| Australian Rules Football | No_1_ Yes_2_ |  |  | |  |  | |  | |  | |
| Soccer | No_1_ Yes_2_ |  |  | |  |  | |  | |  | |
| Basketball | No_1_ Yes_2_ |  |  | |  |  | |  | |  | |
| Cricket | No_1_ Yes_2_ |  |  | |  |  | |  | |  | |
| Netball | No_1_ Yes_2_ |  |  | |  |  | |  | |  | |
| Baseball/softball | No_1_ Yes_2_ |  |  | |  |  | |  | |  | |
| 4 square/ Down ball | No_1_ Yes_2_ |  |  | |  |  | |  | |  | |
| Swimming laps | No_1_ Yes_2_ |  |  | |  |  | |  | |  | |
| Tag/chasey | No_1_ Yes_2_ |  |  | |  |  | |  | |  | |
| Skipping rope | No_1_ Yes_2_ |  |  | |  |  | |  | |  | |
| Martial Arts | No_1_ Yes_2_ |  |  | |  |  | |  | |  | |
| Hockey (field hockey) | No_1_ Yes_2_ |  |  | |  |  | |  | |  | |
| Other (Please state)  **_________________________** | No_1_ Yes_2_ |  |  | |  |  | |  | |  | |

**Question 6 Continued:**

|  |  | **Monday - Friday** | |  | **Saturday** | |  | | **Sunday** | |  |
| --- | --- | --- | --- | --- | --- | --- | --- | --- | --- | --- | --- |
| In the last week which activities did you do? | Did you do this activity? | Number of times | Total Hours/ Minutes | | Number of times | Total Hours/ Minutes | | Number of times | | Total Hours/ Minutes | |
| Roller-blading | No_1_ Yes_2_ |  |  | |  |  | |  | |  | |
| Scooter | No_1_ Yes_2_ |  |  | |  |  | |  | |  | |
| Skateboarding | No_1_ Yes_2_ |  |  | |  |  | |  | |  | |
| Bike riding | No_1_ Yes_2_ |  |  | |  |  | |  | |  | |
| Household chores | No_1_ Yes_2_ |  |  | |  |  | |  | |  | |
| Play on playground equipment | No_1_ Yes_2_ |  |  | |  |  | |  | |  | |
| Play in cubby house | No_1_ Yes_2_ |  |  | |  |  | |  | |  | |
| Bounce on trampoline | No_1_ Yes_2_ |  |  | |  |  | |  | |  | |
| Play with pets | No_1_ Yes_2_ |  |  | |  |  | |  | |  | |
| Walk the dog | No_1_ Yes_2_ |  |  | |  |  | |  | |  | |
| Walk for exercise | No_1_ Yes_2_ |  |  | |  |  | |  | |  | |
| Jogging or running | No_1_ Yes_2_ |  |  | |  |  | |  | |  | |
| Physical Education class | No_1_ Yes_2_ |  |  | |  |  | |  | |  | |
| Sport class at school | No_1_ Yes_2_ |  |  | |  |  | |  | |  | |
| Travel by walking to school  (To and from school = 2 times) | No_1_ Yes_2_ |  |  | |  |  | |  | |  | |
| Travel by cycling to school  ( To and from school = 2 times) | No_1_ Yes_2_ |  |  | |  |  | |  | |  | |
| Other (Please state) | No_1_ Yes_2_ |  |  | |  |  | |  | |  | |

**7. In the past 7 days, during your physical education (PE) classes, how often were you *very active* (playing hard,**

**running, jumping, throwing)? (Tick** √ only one)

| **I don’t do PE……………** |  |
| --- | --- |
| **Hardly ever………………** |  |
| **Sometimes………………** |  |
| **Quite often………………** |  |
| **Always……………………** |  |

**8. In the past 7 days, what did you normally do at *recess* (besides eating food) (Tick √ only one)**

| **Sat down (talking, reading, doing school work)** |  |
| --- | --- |
| **Stood around or walked around** |  |
| **Ran or played a little bit** |  |
| **Ran around and played quite a lot** |  |
| **Ran and played hard most of the time** |  |

**9. In the past 7 days, what did you normally do at *lunch*** (besides eating food) (Tick √ only one)

| **Sat down (talking, reading, doing school work)** |  |
| --- | --- |
| **Stood around or walked around** |  |
| **Ran or played a little bit** |  |
| **Ran around and played quite a lot** |  |
| **Ran and played hard most of the time** |  |

**10. In the past 7 days, on how many days *right after school*, did you do sports, dance or play games in which you were very active? (Tick** √ only one)

| **None………………………** |  |
| --- | --- |
| **1 time last week………...** |  |
| **2 or 3 times last week…** |  |
| **4 or 5 times last week…** |  |
| **6 or 7 times last week…** |  |

**11. In the past 7 days, on how many *evenings***, did you do sports, dance or play games in which you were very active? (Tick √ only one)

| **None………………………** |  |
| --- | --- |
| **1 time last week………...** |  |
| **2 or 3 times last week…** |  |
| **4 or 5 times last week…** |  |
| **6 or 7 times last week…** |  |

**12. *On the last weekend*, how many times did you do sports, dance or play games in which you were very active?**

**(Tick √** only one)

| **None………………………** |  |
| --- | --- |
| **1 time last week………...** |  |
| **2 or 3 times last week…** |  |
| **4 or 5 times last week…** |  |
| **6 or 7 times last week…** |  |

**13. Were you sick last week, or did anything prevent you from doing your normal physical activities? (Circle) YES NO**

**If yes, what prevented you? ____________________________________________________________________**

| **14. Transport *to school* Monday-Friday (Please tick √)** | **Yes** | **No** |  |
| --- | --- | --- | --- |
| a) Did you walk or cycle ***to school today***? |  |  | **If yes complete b and c** |
|  |  |  |  |
| bi) I walked all the way to school |  |  |  |
| bii) I walked to the bus stop or train station |  |  |  |
| biii) I cycled all the way to school |  |  |  |
| b iv) I cycled to the bus stop or train station |  |  |  |

**c) How many minutes did it take you to walk or cycle? _____________ minutes**

| **15. Transport *from school* Monday to Friday (Please tick √)** | **Yes** | **No** |  |
| --- | --- | --- | --- |
| a) Did you walk or cycle ***home from school yesterday***? |  |  | **If yes complete b and c** |
|  |  |  |  |
| bi) I walked all the way |  |  |  |
| bii) I walked home from the bus stop or train station |  |  |  |
| biii) I cycled all the way home |  |  |  |
| biv) I cycled home from the bus stop or train station |  |  |  |

**c) How many minutes did it take you to walk or cycle? _____________ minutes**

**Sedentary Behaviours**

**16. How long did you spend doing the following activities during the last 7 days week?**

*(Think about what you do when you finish school (about 3:30pm) until you go to sleep. This is usually about 6-7 hours per day)*

|  | **Monday - Friday** | **Saturday** | **Sunday** |
| --- | --- | --- | --- |
|  | Total Hours/ Minutes | Total Hours/ Minutes | Total Hours/ Minutes |
| a) Watch TV |  |  |  |
| b) Watch Videos/ DVD’s |  |  |  |
| c) Play video games |  |  |  |
| d) Use a computer for fun (Chat room, internet surfing, games) |  |  |  |
| e) Study or do homework |  |  |  |
| f) Read for fun |  |  |  |
| g) Chat on the phone |  |  |  |
| h) Hang out at home, park or shopping centre (sitting/standing) |  |  |  |
| i) Do hobbies/ crafts |  |  |  |
| j) Music lessons/ practice |  |  |  |
| k)Travel in a car, train, bus or boat/ferry |  |  |  |
| l) Go to church |  |  |  |
| m) Attend Saturday school |  |  |  |
| n) Attend out-of-school-hours tutoring |  |  |  |
