# Supplementary material for: Reliability of the modified child and adolescent physical activity and nutrition survey, physical activity (CAPANS-PA) questionnaire among chinese-australian youth
Source: BMC Med Res Methodol. 2011 Aug 25;11:122. doi: 10.1186/1471-2288-11-122 (PMC3175205; doi:10.1186/1471-2288-11-122)
Supplement: Additional file 2 — Summary of the CAPANS-PA Questionnaire. This file contains a summary of the items contained within the CAPANS-PA questionnaire. The summary outlines the original source of the question item, modifications made to the original CAPANS Year 8, 10 and 11 questionnaire and reliability and validity properties where available. [file 1471-2288-11-122-S2.DOC]

| Additional file 2. Summary of the CAPANS-PA questionnaire* | | | |  |  |  |  | |  | |
| --- | --- | --- | --- | --- | --- | --- | --- | --- | --- | --- |
| **Question** | **Description** | **Source(s)** | **Modifications From CAPANS 2003 8,10 & 11** | | | **Validity** | | **Reliability** | |  |
| Demographic | Students record their:   - Name - Age (Years) - Date of birth - School (Mon-Fri) - Gender (circle) - Postcode of primary residence | CAPANS Years 8, 10 and 11 questionnaire (1-2) | These items were moved to the beginning of instrument instead of being the last questions. Also, language spoken at home and are you an Aboriginal or Torres-Straight Islander was removed. | | |  | |  | |  |
| 1-5.  Country of birth | Students report the country of birth via circling the correct option or adding new country for the following:   - Themselves - Mother and father (if known) - Maternal grandmother and grandfather (if known) | Only birthplace of participant was adapted from CAPANS Years 8, 10 and 11 questionnaire (1-2) | Additions included a list of 10 countries of birth. Also parental and maternal grandparents’ birthplace with the option to write down country of birth. | | |  | |  | |  |
| 6.  Recall previous 7-day activity | Students recall their PA participation during the last 7-days via a list of 32 common physical activities. An additional two ‘other’ activity options can be filled in by students should their activity not appear on the list. Students recall:   - Whether they participated in the last 7-days (Y/N) - Frequency Mon-Fri - Duration Mon-Fri - Frequency Sat & Sun - Duration Sat & Sun | CAPANS Years 8, 10 and 11 questionnaire (1-2)  Adapted from APARQ (3) and CLASS Australian version (4) and Hong Kong version (5) | Reworded to ‘last 7-days’ from ‘usually do during a typical week’ in the CLASS Australian version (4) and ‘last week’ in the Hong Kong CLASS version (5). Removed activity ‘4 square/Down ball’. Added surfing, hockey and martial arts as recommended by CAPANS technical report (1). Also added extra ‘other’ activity item. | | | 29 of the 32 physical activity items are identical to both CLASS surveys. In the Australian CLASS validation, very low correlations were observed between weekly duration of self-reported MVPA and accelerometry data among 10-12y (ρ = -0.04) (4). In contrast, the Hong Kong CLASS validation study (9-12y), correlations between weekly duration of MVPA and accelerometer data were higher for girls than boys (r = 0.48 for girls; r = 0.27 for boys) (5) | | Test-retest reliability (percent agreement) of 28 individual physical activity items ranged from 62 -94% in the Australian CLASS validation study (4). Additionally, intra class correlations (ICCs’) for weekly duration and frequency of MVPA participation among 10-12y participants were higher for frequency than duration (ICC frequency = 0.36; ICC duration = 0.24) (4). In the Hong Kong CLASS validation, acceptable test-retest reliability was observed for duration of weekly MVPA (ICC= 0.71) (5). | |  |

| **QUESTION NUMBER** | **DESCRIPTION** | **SOURCES(S)** | **MODIFICATIONS FROM CAPANS 2003 8, 10 & 11** | **Validity** | **Reliability** |
| --- | --- | --- | --- | --- | --- |
| 7.  Activity perception during PE | Students indicate their perception of how frequently they were very active (playing hard, running, jumping, throwing & not waiting for their turn) during their PE class in the last 7-days by selecting an item from the below checklist:   1. I don’t do PE 2. Hardly ever 3. Sometimes 4. Often 5. Always | CAPANS Years 8, 10 and 11 questionnaire  (1-2)  Adapted from PAQ-C (6) and PAQ-A (7) | None- identical |  |  |
| 8-9. School based activity (Recess & Lunch) | Students indicate the activities conducted at recess and lunch during the past 7-days (aside form eating) by selecting the appropriate item form the below checklist:   1. Sat down (talking, reading, doing school work) 2. Stood or walked around 3. Ran or played a little bit 4. Ran around and played quite a lot 5. Ran and played most of the time | CAPANS Years 8, 10 and 11 questionnaire (1-2)  Originally modified from AHFS 1985 (8) | None- identical |  |  |
| 10-12. Non-school based activity | Students indicate on how many days i) right after school, ii) in the evenings, and on the previous weekend they did sports, dance or play games in which they were very active by selecting the appropriate item from the below checklist:   1. None 2. 1 time last week 3. 2 or 3 times last week 4. 4 or 5 times last week 5. 6 or 7 times last week | CAPANS Years 8, 10 and 11 questionnaire (1-2)  Adapted from PAQ-C (6) and PAQ-A (8) | None- identical |  |  |
| 13.  Prevented from normal activity | Students indicate if they were prevented from engaging in their normal activities in the previous week (Yes/No) and for what reason e.g. Injured, Flu, School camp | CAPANS Years 8, 10 and 11 questionnaire (1-2) | None- identical |  |  |
| 14-15.  Active transport to and from school | Students recall how they travelled to school and from school for the Friday just passed by selecting the appropriate items from the below checklist and open ended item:   1. Did you walk or cycle to/from school on Friday (Y/N)   bi) Did you walk all the way to school/home from school (Y/N)  bii) Did you walk to school/home from school from the bus/train station (Y/N)  biii) Did you cycle all the way to school/ home from school (Y/N)  biv) Did you cycle to school/ home from school from the bus/train station (Y/N)  c) How many minutes did it take to walk/cycle to and from school? (Record in min) | CAPANS Years 8, 10 and 11 questionnaire (1-2)  Adapted from AHFS 1985 (8) | None- identical |  |  |
| 17.  Recall previous 7-day sedentary behaviour | Students recall their sedentary behaviours during the last 7-days via a list of 14 common sedentary activities. Students recall:   - Duration of participation Mon-Fri - Duration of participation Sat & Sun | CAPANS Years 8, 10 and 11 questionnaire (1-2)  CLASS Australian version (4), Hong Kong version (5) and APARQ (3) | Reworded to ‘last 7-days’ from ‘Usually spend doing on an average school day and weekend’. Added DVD to ‘Watch Videos’ item. Removed ‘extra tutoring’ from ‘Study or do homework’ and ‘Saturday school’ from ‘Go to church’ and created separate items for ‘Attend Saturday school’ and ‘Attend out-of-school hours tutoring’. | In the CLASS Hong Kong validation study, duration spent in weekly sedentary activities showed higher correlations with accelerometer derived data for girls than compared to boys (r= 0.06 boys, r = 0.25 girls) (5). | Test-retest reliability of self-reported duration spent in weekly sedentary activities was (ICC = 0.69) in the Hong Kong CLASS validation (5). |

* The authors would like to note that the above information is correct **to the best of our knowledge**; it is likely that the development of the CAPANS-PA questionnaire has taken inspiration from many physical activity self-report questionnaires among children, adolescents and adults. In the interest of openness, the authors have tried to clearly indicate where possible the source of the questionnaire items from the original CAPANS Year 8, 10 and 11 questionnaire. This list may not be exhaustive and should be utilised with caution.

**References**

1. Hands B, Parker H, Glasson C, Brinkman S, Read H: **Results of Western Australian Child and Adolescent Physical Activity and Nutrition Survey 2003 (CAPANS). Physical Activity Technical Report.** (WA) Government of Western Australia, Department of Sport and Recreation; 2004.
2. McCormack G, Giles-Corti B: **Report to the Physical Activity Taskforce, Evaluation and Monitoring Group: An assessment of self-report questionnaires and motion sensors for measuring physical activity in children.** pp. 87: Department of Public Health, The University of Western Australia; 2002:87.
3. Booth M, Mackaskill P, McLellan L, Phongsavan P, Okely T, Patterson J, Wright J, Bauman A, Baur L: **NSW Schools Fitness and Physical Activity Survey.** (NSW Department of School Education ed.: NSW Department of School Education; 1997.
4. Telford A, Salmon J, Jolley D, Crawford D: **Reliability and validity of physical activity questionnaires for children: the Children's Leisure Activities Study Survey (CLASS).** *Pediatr Exerce Sci* 2004, **16:**64-78.
5. Huang YJ, Wong SH, Salmon J: **Reliability and validity of the modified Chinese version of the Children's Leisure Activities Study Survey (CLASS) questionnaire in assessing physical activity among Hong Kong children.** *Pediatr Exerc Sci* 2009, **21:**339-353.
6. Crocker PR, Bailey DA, Faulkner RA, Kowalski KC, McGrath R: **Measuring general levels of physical activity: preliminary evidence for the Physical Activity Questionnaire for Older Children.** *Med Sci Sports Exerc* 1997, **29:**1344-1349.
7. Kowalski K, Crocker P, Kowalski N: **Convergent Validity of the Physical Activity Questionnaire for Adolescents.** *Pediatrc Exerc Sci* 1997, **9:**342-352.
8. ACHPER Publications: **Australian Health and Fitness Survey 1985. The Fitness, Health and Physical Performance of Australian School Students Aged 7-15 Years.** ACHPER Publications; 1985.
